# The Impact of Various Organic Phosphorus Carriers on the Uptake and Use Efficiency in Barley

**DOI:** 10.3390/ijms242417191

**Published:** 2023-12-06

**Authors:** Yuanfeng Huo, Jingyue Wang, Yinggang Xu, Deyi Hu, Kexian Zhang, Bingjie Chen, Yueyi Wu, Jiaxin Liu, Tianlang Yan, Yang Li, Chaorui Yan, Xuesong Gao, Shu Yuan, Guangdeng Chen

**Affiliations:** College of Resources, Sichuan Agricultural University, Chengdu 611130, China; huoyf9918@163.com (Y.H.); wjyue123@163.com (J.W.); xuyinggang2021@126.com (Y.X.); hudeyi18@126.com (D.H.); 13699492269@139.com (K.Z.); 15528133002@163.com (B.C.); wyy06302022@163.com (Y.W.); 18982098981@163.com (J.L.); 18382873088@163.com (T.Y.); yangli922@yahoo.com (Y.L.); yanchaorui@sicau.edu.cn (C.Y.); gxs80@126.com (X.G.)

**Keywords:** organic phosphorus, rhizosphere, phosphorus utilization efficiency, genotypic difference, gene expression

## Abstract

Organic phosphorus (OP) is an essential component of the soil P cycle, which contributes to barley nutrition after its mineralization into inorganic phosphorus (Pi). However, the dynamics of OP utilization in the barley rhizosphere remain unclear. In this study, phytin was screened out from six OP carriers, which could reflect the difference in OP utilization between a P-inefficient genotype Baudin and a P-efficient genotype CN4027. The phosphorus utilization efficiency (PUE), root morphological traits, and expression of genes associated with P utilization were assessed under P deficiency or phytin treatments. P deficiency resulted in a greater root surface area and thicker roots. In barley fed with phytin as a P carrier, the APase activities of CN4027 were 2–3-fold lower than those of Baudin, while the phytase activities of CN4027 were 2–3-fold higher than those of Baudin. The PUE in CN4027 was mainly enhanced by activating phytase to improve the root absorption and utilization of Pi resulting from OP mineralization, while the PUE in Baudin was mainly enhanced by activating APase to improve the shoot reuse capacity. A phosphate transporter gene *HvPHT1;8* regulated P transport from the roots to the shoots, while a purple acid phosphatase (PAP) family gene *HvPAPhy_b* contributed to the reuse of P in barley.

## 1. Introduction

P is absorbed by plants only as Pi, which is one of the least accessible macronutrients [1]. The productivity of plants is often considered to be limited by P availability [2]. The proportion of Pi in the soil P pool is very low, while OP represents even up to 50–70% of the total P in mineral soils [3,4]. The main OP forms include monophosphate, diphosphate and organic polyphosphate [2]. Inositol P is the most abundant form of OP in soil, accounting for more than half of the total OP content, which can be accompanied with Ca^2+^, Mg^2+^ and other alkaline soil ions [5]. Phytin, C_6_H_6_(OPO_3_H_2_)_6_, is the hexaphosphoric acid ester of the hexavalent alicyclic alcohol mesoinositol [6]. Phytin is relatively unavailable, since it is strongly adsorbed by soil and immune to degradation by soil microbes [7]. However, it can be absorbed and utilized by crops via the hydrolysis of APase and phytase [8,9]. Similarly, the Pi released by OP mineralization is rapidly adsorbed by the soil to form water-insoluble minerals, of which only a small fraction is available to plants [10].

Barley have evolved physiological mechanisms such as changing its root morphology, releasing root exudates and contacting rhizosphere microorganisms to enhance its ability to acquire P in soils with low P availability [11]. The accumulation of more photosynthates promotes root growth, increases the number of primary and secondary roots and increases the length of root hairs, which are typical root morphological responses of plants to P deficiency [12]. In addition, plant roots will secrete more H^+^, organic acids and phosphatases, change the pH of the rhizosphere environment and release P adsorbed on the surface of soil minerals to improve their OP uptake from the soil solution [13]. When plants sense the signal of P deficiency from the external environment, they will induce the secretion of APase and degrade OP into Pi [14]. APase can be divided into intracellular acid phosphatase (IAP) and secretory acid phosphatase (SAP) according to whether it is secreted into the extracellular cell. Bozzo et al. [15] demonstrated that, under the condition of P deficiency, plants utilize intracellular OP preferentially, and only exocytic APase will be secreted when intracellular P homeostasis cannot be maintained.

Purple acid phosphatases (PAPs) are a family of special APase, which can catalyze the hydrolysis of monophosphate ester and anhydride organic matter at an acidic pH, and release the corresponding alcohol and Pi [16]. In plants, many members of the PAP gene family have been identified due to the fact that they play key roles in plant adaptation to P deficiency [17,18]. Wang et al. [19] demonstrated that *GmPAP12* in soybean may promote the utilization of P via the soybean root nodules under low P stress, which was reflected by the significant increase in APase and phytase activities in mature soybean root nodules. More Arabidopsis PAPs have been characterized compared with other species. Of the 29 verified Arabidopsis PAP genes, *AtPAP10* expression is induced by local and systemic Pi starvation signals in the leaves and roots [20].

Since P is a non-renewable resource, but OP accounts for a large proportion in the total P of soil, both of which are limited, improving the utilization rate of the soil’s OP could provide a guarantee for sustainable agricultural development. We have collected the barley cultivation varieties Baudin (a high-yield malt variety), Franklin (a double-row malt barley) and Grimmet from the main barley producing areas in Australia; a wild barley variety CN4027 planted in rainfall planting areas in western Australia and a Japanese local variety CN4079. In our previous study [21], a one-year evaluation of phosphorus absorption efficiency was performed. And we found that the wild variety CN4027 and the cultivated variety Baudin showed the greatest differences in organic phosphorus utilization. A recombinant inbred line (RIL) population of 128 crosses between the variety Baudin (a P-inefficient genotype) and the variety CN4027 (a P-efficient genotype) was generated to detect a quantitative trait locus (QTLs) for PUE traits [21]. In this study, we evaluated the growth of barley under diverse OP nutrition conditions, the difference in P utilization capacity of two barley genotypes (Baudin and CN4027) under different concentrations of OP treatments, as well as their influence on the expression levels of key genes related to APase and phytase activities.

## 2. Results

### 2.1. The Growth of Barley under Diverse OP Treatments

**Figure 1:**
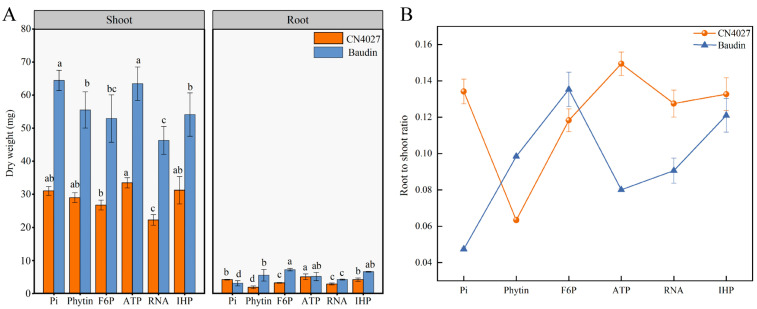
Shoot biomass, root biomass (**A**) and root-to-shoot ratio (**B**) under different OP treatments. Different letters behind the values indicate significant differences among organic phosphorus treatments within each genotype at the 0.05 level.

The biomass and total root morphology of the two genotypes of barley were influenced by different OP supplies: Pi (a control treatment), phytin, fructose-6-phosphate (F6P), ATP, RNA and inositol hexaphosphate (IHP) (Figure 1 and Table 1). Regardless of the OP treatments, the biomass of Baudin was higher than that of CN2047 (Figure 1A). The shoot biomass in the medium with ATP as the only P source was significantly higher than the other treatments (*p* < 0.05; Figure 1A), while the lowest shoot biomass was recorded under RNA treatment. The highest root biomass of CN4027 was observed in ATP treatment, while the highest root biomass of Baudin was observed in F6P treatment. The root-to-shoot ratio can reflect the distribution of dry matter in plants. As shown in Figure 1B, the root-to-shoot ratio of genotype CN4027 under Pi was the highest. The root-to-shoot ratios of CN4027 were higher than those of Baudin, except for with phytin and F6P.

The response of root morphology to different OP supplies was not always the same between the two cultivars. ATP treatment promoted the growth of the total root length of CN4027, while the total root length of Baudin was longer under the ATP or F6P treatments (Table 1). The total root length of either CN4027 or Baudin was significantly decreased by IHP or Pi treatment (*p* < 0.05). Similar results were found for the root volume and root surface area. Moreover, the changing degrees of primary root length of the two barley genotypes in response to the OP sources were different (Table 2). Compared with other OP treatments, the primary root length, root surface area and root volume of CN4027 were higher under Pi treatment. However, these parameters dramatically declined in Baudin under Pi treatment.

The lateral root length and root surface area of CN4027 under ATP treatments were higher than those under the other treatments (192% higher than that under the IHP treatment; Table 3). Differently, the lateral roots of Baudin grew best with either ATP or F6P application. The genotype CN4027 was found with the smallest root surface area in the IHP treatment (1.60 cm^2^), while the smallest root surface area of the genotype Baudin appeared under the treatment of RNA (1.84 cm^2^).

In general, the two genotypes of barley grew best under ATP supply. However, according to the root dry weight, the largest difference between two genotypes was found with the phytin treatment (Figure 1A). Phytin was then selected as the exogenous OP source for the subsequent experiments to study the P utilization capacity between the two barley genotypes under different P concentrations.

### 2.2. The Growth of Barley Nourished with Different Concentrations of Phytin

**Figure 2:**
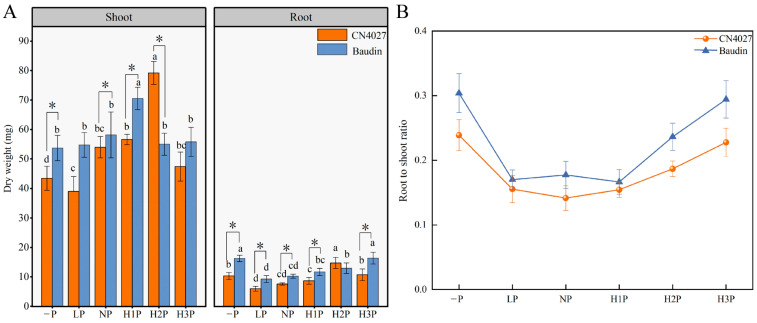
Shoot biomass, root biomass (**A**) and root-to-shoot ratio (**B**) under different levels of phytin treatments. −P, no phytin; LP, 0.625 mM phytin; NP, 1.25 mM phytin; H1P, 2.5 mM phytin; H2P, 3.75 mM phytin; H3P, 5 mM phytin. Different letters behind the values indicate significant differences among different levels of phytin treatments within each genotype at the 0.05 level. “*” indicates significant differences at the 0.05 level between the two genotypes, for the same phytin treatment.

The biomass of the two genotypes of barley was significantly different with the change in phytin concentrations (*p* < 0.05; Figure 2A). Six levels of phytin, 0 mM (−P), 0.625 mM (LP), 1.25 mM (NP), 2.5 mM (H1P), 3.75 mM (H2P) and 5 mM (H3P), were applied, respectively. The shoot biomass increased with the increase in phytin concentration. The shoot biomass of CN4027 reached its maximum in H2P, while that of Baudin reached its maximum in H1P (Figure 2A). The root biomass of Baudin was 33.94–62.93% higher than CN4027 except in H2P (Figure 2A). Meanwhile, the root-to-shoot ratio of the two genotypes was narrower under the “−P” condition. The ratio decreased after the supply of phytin and then increased with the increase in phytin concentration (Figure 2B). In general, the total root length of the two barley genotypes was increased by the phytin treatments (Table 4). The total lengths of the Baudin roots were 7.28% to 50.59% higher than those of CN4027. The highest root length of barley was recorded under H3P (80.74 cm for CN4027; 121.59 cm for Baudin) and the lowest was recorded under NP (36.92 cm for CN4027; 50.86 cm for Baudin).

### 2.3. Utilization of P in Barley Nourished with Different Concentrations of Phytin

**Figure 3:**
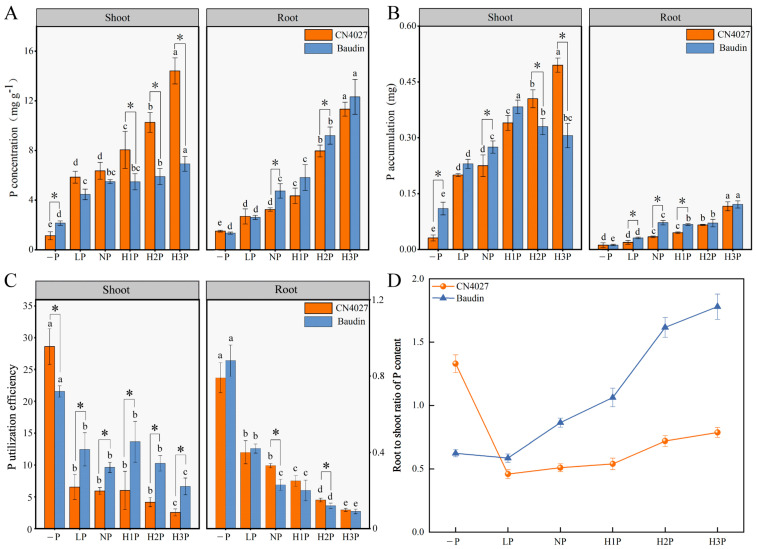
Shoot P concentration and root P concentration (**A**), shoot P accumulation and root P accumulation (**B**), shoot P utilization efficiency and root P utilization efficiency (**C**) and root-to-shoot ratio of P content (**D**) of CN4027 and Baudin under −P, LP, NP, H1P, H2P or H3P treatment. Different letters behind the values indicate significant differences among different levels of phytin treatments within each genotype at the 0.05 level. “*” indicates significant differences at the 0.05 level between the two genotypes, for the same phytin treatment.

With the increase in phytin concentration, the P content increased significantly in both CN4027 and Baudin (*p* < 0.05; Figure 3). Regardless of the phytin concentrations, the shoot P contents of CN4027 were always higher than Baudin, except for the “−P” treatment, but the root P contents showed the opposite trend so that CN4027’s levels were always lower than Baudin’s (Figure 3A). The P accumulation levels of the two genotypes of barley increased with the increase in phytin concentration on the whole, but the shoot P accumulation in Baudan reached the turning point at H1P treatment and then declined (Figure 3B). After 3 weeks of “−P” treatment, the phosphorus utilization efficiency (PUE) of both Baudin and CN4027 significantly increased (Figure 3C); however, it decreased with high levels of phytin. According to Figure 3D, CN4027 had the highest proportion of P content in its roots under the “−P” treatment, while the highest root-to-shoot ratio of P content in Baudin was recorded under H3P treatment.

### 2.4. Acid Phosphatase Activity and Phytase Activity of Barley

**Figure 4:**
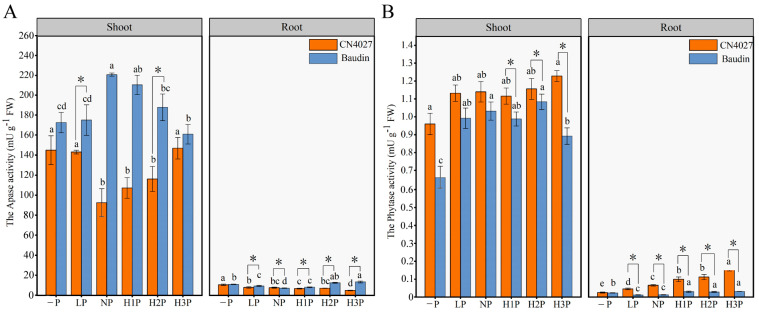
Shoot and root APase activities (**A**) and shoot and root phytase activities (**B**) under different levels of phytin treatments. Different letters behind the values indicate significant differences among different levels of phytin treatments within each genotype at the 0.05 level. “*” indicates significant differences at the 0.05 level between the two genotypes, for the same Phytin treatment.

The APase activities and phytase activities of the two genotypes of barley were measured, and it was found that the APase activities in Baudin were higher than those in CN4027, which was contrary to the measured results of phytase activities (Figure 4). Baudin showed 41.61% to 91.19% higher APase activities when compared with CN4027, and the biggest difference was found at NP treatment (Figure 4A). Meanwhile, with an increase in the exogenous phytin levels, Baudin’s APase activities increased first and then declined, while the change trend for CN4027 was the opposite (Figure 4A). Compared to the “−P” condition, the shoot phytase activities of the two genotypes were enhanced by 10.41% to 20.92% (in CN4027) and 33.49% to 62.23% (in Baudin) under the phytin treatments, respectively (Figure 4B). In general, the shoot phytase activities of CN4027 were significantly higher than those of Baudin, and the greatest difference was found under the “−P” condition. The root phytase activities of the two genotypes of barley increased with the increase in phytin concentration, and the increases in CN4027 were higher than those of Baudin, with the highest increases of 512.99% and 36.59%, respectively (Figure 4B). These results indicated that different genotypes of barley may enhance their adaptability to low P stress and the utilization efficiency of phytin by increasing their shoot APase activities (in Baudin) or root phytase activities (in CN4027).

### 2.5. Phylogenetic Analysis of PAPs and PHTs in Barley

**Figure 5:**
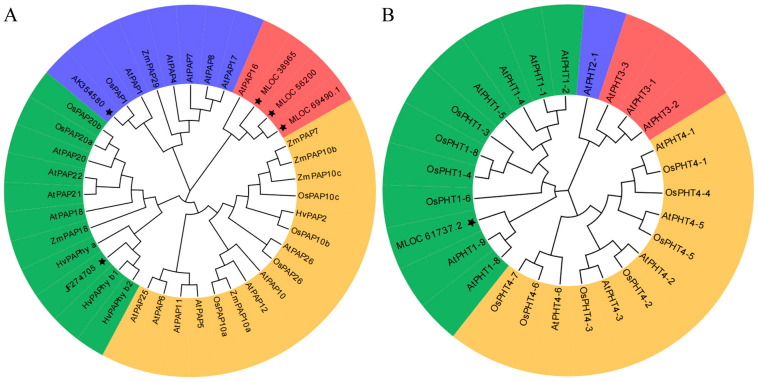
The phylogenetic tree of PAPs (**A**) and PHTs (**B**) from Arabidopsis, rice, maize, wheat and barley was constructed using the MEGA 7.0 software and using the neighbor-joining method. Bootstrapping (1000 replicates) was used to evaluate the degree of support for each group in the phylogenetic tree. At: *Arabidopsis thaliana*; Os: *Oryza sativa*; Ta: *Triticum aestivum*; Zm: *Zea mays*; Hv: *Hordeum vulgare*. The “Star” indicates a MLOC protein, which was renamed in this study. Different background colors mark different clusters.

To further study the phylogenetic relationship of the PHT and PAP protein families in barley, a neighbor-joining (NJ) phylogenetic tree was constructed using MEGA 7.0. A cluster analysis of the PAP proteins and PHT proteins from Arabidopsis, barley, wheat (*Triticum aestivum*), maize (*Zea mays*) and rice (*Oryza sativ*) is illustrated in Figure 5. Either PAPs or PHTs may be divided into four different groups: Groups I, II, III and IV. Based on the cluster analysis, HvPAPhy_b was related in close kinship to the PAP I-2 family member HvPAPhy_a, and MLOC_61737 was highly similar to AtPHT1;8 and AtPHT1;9, which are members of the PHT1 family; thus, MLOC_61737 was renamed HvPHT1;8. Similarly, based on the results of the cluster analysis, the four candidate genes AK354580, MLOC_38965, MLOC_56200 and MLOC_69490 were renamed HvPAP1, HvPAP16;1, HvPAP16;2 and HvPAP16;3, respectively.

### 2.6. Expression of P-Utilization-Related Genes

**Figure 6:**
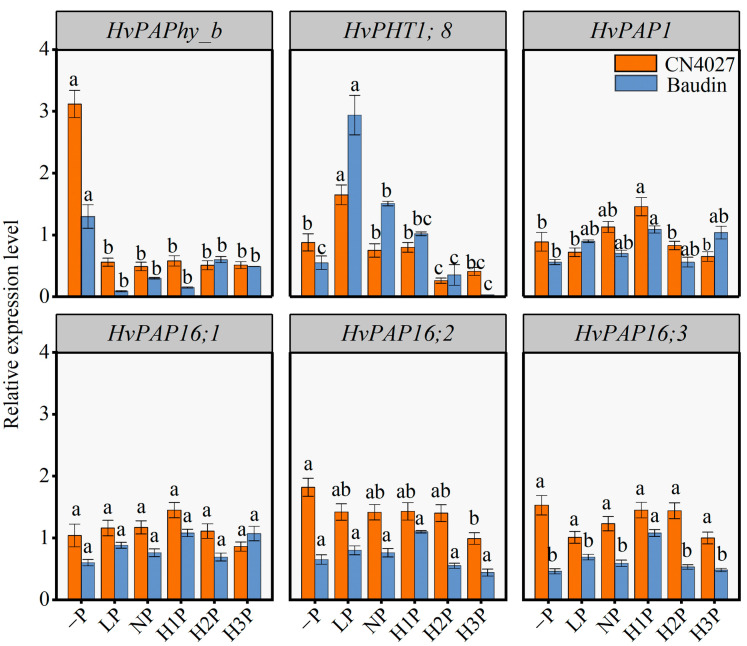
Relative expression levels of *HvPAPhy_a*, *HvPHT1;8*, *HvPAP1*, *HvPAP16;1*, *HvPAP1;2* and *HvPAP16;3* in barley treated with different levels of phytin. Different letters behind the values indicate significant differences among different levels of phytin treatments within each genotype at the 0.05 level.

The relative expression levels of six genes potentially regulating P utilization processes in barley under different levels of phytin treatments were determined. CN4027 and Baudin showed a similar expression pattern (Figure 6). LP treatment dramatically upregulated the expression of *HvPHT1;8*, while the expression of *HvPAPhy_b* was most highly upregulated under the “−P” condition. Moreover, the NP and H1P treatments significantly upregulated *HvPAP1* expression compared with the “−P” condition (*p* < 0.05). However, there was no significant difference in the relative expression of the genes *HvPAP16;1* and *HvPAP16;3* in barley treated with different concentrations of phytin.

## 3. Discussion

Soil P is mainly absorbed and utilized by plant roots in the form of Pi. However, the OP in soil also has great potential for P supply. Most plants in nature are under low P conditions [22]. Therefore, it is necessary to evaluate the ability of plants to utilize different OP sources in soils. Relevant genetic improvement strategies will alleviate the P deficiency in crop plants, promote plant growth and development and increase crop yields [23,24,25].

Plants reflect their utilization capacity for different P sources by changing their biological traits, especially their biomass [24]. The higher dry weight of Duo grass (*Duo festulolium*) [26], wheat (*Triticum aestivum* L.) [27] and ryegrass [28] recorded under ATP treatment were attributed to the more developed root systems of the plants, which improved their P acquisition efficiency (PAE) [29]. In the current study, the maximum total root length and root surface area parameters of barley appeared under ATP, indicating that the low ATP environment was the most restrictive to barley roots. However, barley under IHP treatment had a lower biomass, which was maybe because of its own chemical characteristics since IHP has six phosphate groups and is usually highly binding and stable, making it more difficult to desorption than other OP [30,31]. Among all six OP treatments, the largest difference in the root-shoot ratio between Baudin and CN4027 was recorded under the phytin treatment. This phenomenon suggested that the cultivated variety Baudin responds to phosphorus restriction by enhancing shoot growth, while the wild variety CN4027 responds to phosphorus restriction by promoting root growth. And phytin, which could best reflect the genotype difference between CN4027 and Baudin, was selected as the exogenous OP source for the subsequent experiments.

When plants suffer from P starvation, they will increase the available P in the environment by extending their root length and growing more root hairs, secreting organic substances such as organic acids, APase and phytase, which can catalyze monophosphate esters into available P [32]. The root morphology of P-sensitive plants varies more dramatically under low P conditions [8,9]. CN4027 was more effective in inducing root elongation under the “−P” conditions. However, the total root length of Baudin increased mainly under F6P treatment, but not under the “−P” conditions, maybe indicating that Baudin was relatively insensitive to the P starvation conditions. All these data demonstrated that the root system of CN4027 was more sensitive to low P stress than Baudin.

P deprivation restricts the growth of the main roots in barley, and meanwhile induces the lateral roots’ elongation with higher numbers of root hairs. This developmental regulation leads to a higher ratio of roots to shoots and changes the root architecture significantly for greater nutrient uptake, which is in line with earlier reports [13,33]. The roots of CN4027 and Baudin grown in “−P” medium secreted more APase, and the roots of CN4027 secreted more APase than Baudin under the same phytin concentration. The APase and phytase activities were also enhanced by H2P and H3P treatments (3.75 mM and 5 mM Phytin), because the release of these enzymes from the plant roots is influenced by the P requirements of plants [34]. Comparing the P contents of the two genotypes of barley, it could be found that the wild variety CN4027 was more adaptable to an environment with phytin as the P carrier. The APase activity of the shoots in Baudin was higher than that of CN4027, which may be one of the reasons for the higher PUE in the shoots of Baudin than those of CN4027. Moreover, the root phytase activity of CN4027 was higher than that of Baudin, resulting in the higher root PUE of CN4027 than that of Baudin. In a nutshell, the PUE in CN4027 was mainly enhanced by activating phytase to improve the root absorption and utilization of Pi resulting from OP mineralization, while the PUE in Baudin was mainly enhanced by activating APase to improve the shoot reuse capacity. Meanwhile, the increase in phytase activity contributed more to the increase in the shoots’ P content than APase.

Different P levels affected gene expression and related enzyme activities [35], and then affected the plant phenotype and P utilization capacity [36]. The relative expression levels of phytin-utilization-related genes was further explored. The relative expression levels of a phosphate transporter gene *HvPHT1;8* and a purple PAP family gene *HvPAPhy_b* were significantly affected by different concentrations of phytin (*p* < 0.05; Figure 5). The PHT1 family of Pi transporters mediate P uptake and re-mobilization in plants [36,37]. The expression of *HvPAPhy_b* was induced by low P stress, which was mainly expressed during germination, flowering and grain formation, which contributed to the reuse of P in plants and improved the shoot PUE of barley [38]. Another report on Arabidopsis showed that both *AtPHT1;8* and *AtPHT1;9* were involved in the intercellular transport of Pi [39]. Here, we correlated the root-to-shoot ratio of P content with the expression level of *HvPHT1;8* and indicated that the gene *PHT1;8* was related to P transport from the roots to the shoots. In summary, *HvPAPhy_b* was involved in root growth and shoot P utilization under the “−P” conditions, while *HvPHT1;8* regulated the primary root growth and P transport from the roots to the shoots in barley under LP treatment.

## 4. Materials and Methods

To study the response of two barley varieties to different OP, we conducted two relatively independent but closely connected experiments to investigate OP species and their unique utilization patterns, followed by screening the optimal OP concentrations for barley growth.

### 4.1. Plant Materials

Two genotypes of barley were used for this study: CN4027, a wild barley (*Hordeum spontaneum*) P-efficient genotype, and Baudin, a cultivated barley (*H. vulgare*) P-inefficient genotype [21,40].

### 4.2. Experimental Design

**Experiment #1**

Baudin and CN4027 were grown in MS culture medium treated with different OP. Phytin, ATP, RNA, fructose-6-phosphate (F6P) and inositol hexaphosphate (IHP) were used at concentrations of 1.25 mM, and 1.25 mM Pi treatment was used as a control. There were three biological replicates for each treatment.

Consequently, 30 seeds of each fully grown and consistently tested barley sample were washed once with alcohol and sterile distilled water, treated with 0.05% HgCl_2_ containing trace HCl for 2 min and finally washed 8 times in sterile distilled water to remove all sterilization agents. The sterilized seeds were germinated in the dark for 2 d with wet washed river sand in a nursery seedling tray. When the roots were 2–3 cm long, three of the uniformly germinated seeds of each genotype were washed and transplanted into the MS medium. Per liter of dephosphorized MS medium, it contained 20.62 mM NH_4_NO_3_, 18.79 mM KNO_3_, 2.99 mM CaCl_2_, 1.5 mM MgSO_4_, 1.25 mM K_2_SO_4_, 5 μM KI, 100 mM H_3_BO_3_, 92.5 μM MnSO_4_, 30 μM ZnSO_4_, 1 μM NaMoO_4_, 0.1 μM CoCl_2_, 0.1 μM CuSO_4_, 100 μM Fe-EDTA, 0.55 μM myoinositol, 4.1 μM nicotinic acid, 2.4 μM pyridoxine HCl, 0.3 μM thiamine HCl, 30 μM Glycine, 10 g Sucrose, 5.1 g MES and 10 g Agar. NaOH/H_2_SO_4_ was used to keep the pH at 6.5. Plants in the medium were kept in an illumination incubator with a 16/8 h (light/dark) cycle at 20 ± 2 °C and 70% relative humidity.

**Experiment #2**

Phytin was selected from **Experiment #1**, which could reflect the genotype variation and was suitable for barley growth. The six levels of phytin were 0 mM (−P), 0.625 mM (LP), 1.25 mM (NP), 2.5 mM (H1P), 3.75 mM (H2P) and 5 mM (H3P), respectively. The seeds were sterilized and transplanted into the dephosphorized MS medium according to **Experiment #1**.

### 4.3. Analysis of Plant Growth

The shoots and roots were harvested separately at 3 weeks after treatment, and the harvested tissues were dried at 105 °C for 30 min followed by 75 °C to a constant weight. The root morphology was measured using a root scanner. The root was scanned digitally first (Epson Perfection V700 Photo, Nagano, Japan), and then the total root length, total root surface area, total root volume, average diameter and number of root tips were analyzed using the WinRHIZO Pro V2007d (Regent Instrument Inc., Quebec, ON, Canada).

### 4.4. Determination of P Content and Enzyme Activity

The P content in the shoots and roots of barley under different treatments was determined using a phosphomolybdate colorimetric assay, as described previously [40]. The total P uptake at each sampling part was calculated based on the plant’s dry weight and the P concentration in the tissues. As described by Deng et al. [40] and Guo et al. [41], the shoot PUE was calculated as the shoots’ dry weight per unit of P in the shoots. The pNPP was used to quantify the Apase activity [42]. After sample collection, the phytase activity in the plants was determined using the protocols described by Mohsin et al. [7].

### 4.5. Phylogenetic Analysis

Based on our previous analysis of the major quantitative trait locus controlling the PUE in barley under phytic acid treatment [21], the molecular marker sequences among QTLs were compared with WGSMorex whole-genome sequencing data in © Leibniz Institut (IPK)’s Barley Blast Server Database. And then, the candidate genes related to P utilization traits in barley were screened out preliminarily, which were *AK354580*, *MLOC_38965*, *MLOC_56200*, *MLOC_69490* and *MLOC_61737* [21]. In addition, the reported barley phytase gene *HvPAPhy_b* [43] was also analyzed here.

On the basis of multiple sequence alignments, a neighbor-joining tree of PAPs and PHTs was constructed using the MEGA 7.0 software. The complete deletion was used to deal with gaps or missing data in sequences, and the distance between sequences was estimated after Poisson correction. Additionally, evolutionary trees were improved using the Evolview software (https://evolgenius.info//evolview-v2/#login, accessed on 6 January 2023).

### 4.6. Real-Time PCR

The total RNA was extracted from the leaves of the two genotypes of barley using RNAprep Pure Polysaccharide Polyphenol Plant Total RNA Extraction Kit (Tiangen, Beijing, China) [44]. Agarose gel of 1% concentration was prepared, electrophoresis was performed using the JY200C electrophoresis apparatus (Beijing JUNYI Electrophoresis Co., Beijing, China) and the detection was performed using the Bio-Rad Gel Imaging System (Bio-Rad., Hercules, CA, USA). The cDNA was synthesized from 1 mg of the total RNA using random (dT). The primers were designed using the Primer 6.0 software. The qRT-PCR was performed on the LightCycler 480 machine (Bio-Rad., CA, USA) according to the instructions of the manufacturer. The transcript levels were calculated relative to *Hv_GAPDH* using the formula 2^−∆Ct^. The primers are listed in Table S1.

## 5. Conclusions

This study has provided evidence of the genotypic control of the PUE for two barley cultivars. The molecular mechanism underlying the PUE difference between the two cultivars has been proposed. Baudin mainly realizes the redistribution of P in vivo by increasing the activity of APase, while CN4027 utilizes phytase. The expression levels of *HvPHT1;8* and *HvPAPhy_b* were significantly regulated by the concentration of phytin. *HvPHT1;8* regulated the primary root growth and P transfer from the roots to the shoots under low P treatment. *HvPAPhy_b* contributed to the reuse of P in plants and improved the shoot PUE of barley.

## Figures and Tables

**Table 1:** Total root morphology under different OP supplies.

OP	Length (cm)		Surface Area (cm^2^)		Average Diam. (mm)		Volume (mm^3^)	
	CN4027	Baudin	CN4027	Baudin	CN4027	Baudin	CN4027	Baudin
Pi	29.5 ± 0.6c	19.3 ± 1.3d	4.23 ± 0.50bc	4.24 ± 0.68e	0.38 ± 0.01a	0.38 ± 0.01bc	40.3 ± 3.9b	24.5 ± 4.5c
Phytin	30.8 ± 0.1c	49.5 ± 2.9b	3.72 ± 0.32c	7.00 ± 0.73bc	0.36 ± 0.03a	0.44 ± 0.02b	36.0 ± 6.0b	58.5 ± 8.5b
ATP	42.2 ± 3.6b	60.2 ± 3.7ab	5.43 ± 0.33ab	9.37 ± 0.33a	0.40 ± 0.03a	0.35 ± 0.02c	23.5 ± 0.5c	57.8 ± 1.0b
RNA	31.9 ± 1.5c	27.1 ± 5.1cd	3.34 ± 0.39cd	4.85 ± 0.46de	0.37 ± 0.04a	0.72 ± 0.02a	34.0 ± 3.2bc	84.3 ± 6.8a
F6P	33.6 ± 3.7c	63.8 ± 3.2a	4.02 ± 0.57c	8.79 ± 0.48a	0.38 ± 0.02a	0.44 ± 0.03b	43.5 ± 2.5b	89.5 ± 8.5a
IHP	18.1 ± 0.3d	53.3 ± 4.1ab	2.35 ± 0.16d	7.99 ± 0.80ab	0.39 ± 0.04a	0.45 ± 0.03b	24.7 ± 3.4c	95.7 ± 8.0a

Values are means of three biological replicates (SE). Statistical differences (*p* < 0.05) between OP treatments are indicated by different letters in each column.

**Table 2:** Primary root morphology under different OP supplies.

Treatment	Length		Surface Area		Volume	
	CN4027	Baudin	CN4027	Baudin	CN4027	Baudin
Pi	4.68 ± 0.36b	2.97 ± 0.40c	1.06 ± 0.09a	0.63 ± 0.16c	17.7 ± 1.4a	11.8 ± 2.6e
Phytin	3.92 ± 0.23c	10.3 ± 0.1b	0.68 ± 0.14c	3.35 ± 0.52ab	12.4 ± 1.7b	42.0 ± 5.6bc
ATP	3.29 ± 0.05d	10.6 ± 0.5b	0.76 ± 0.01bc	2.92 ± 0.24ab	17.1 ± 0.5a	31.9 ± 2.8d
RNA	3.95 ± 0.30c	11.0 ± 1.1b	0.51 ± 0.06d	3.01 ± 0.58ab	13.8 ± 0.4ab	47.7 ± 3.0ab
F6P	2.83 ± 0.22d	13.9 ± 0.9a	0.74 ± 0.09bc	3.70 ± 0.35a	13.6 ± 1.2ab	58.0 ± 4.6a
IHP	3.41 ± 0.03cd	14.5 ± 0.2a	0.75 ± 0.06bc	3.78 ± 0.27a	12.3 ± 2.6b	57.9 ± 5.7a

Values are means of three biological replicates (SE). Statistical differences (*p* < 0.05) between OP treatments are indicated by different letters in each column.

**Table 3:** Lateral root morphology under different OP supplies.

Treatment	Length		Surface Area		Volume	
	CN4027	Baudin	CN4027	Baudin	CN4027	Baudin
Pi	24.8 ± 0.2b	16.3 ± 0.9cd	3.17 ± 0.56c	3.55 ± 0.52c	22.6 ± 2.6c	12.7 ± 1.9d
Phytin	26.8 ± 0.1b	39.3 ± 2.8b	3.04 ± 0.18c	3.65 ± 0.21c	23.6 ± 4.3bc	16.5 ± 2.9d
ATP	38.9 ± 3.6a	49.6 ± 3.2a	4.67 ± 0.34a	6.45 ± 0.09a	26.4 ± 4.6d	26.0 ± 1.7c
RNA	27.9 ± 1.3b	16.1 ± 4.0d	2.88 ± 0.33c	1.84 ± 0.33d	20.2 ± 2.9c	36.7 ± 3.9ab
F6P	30.8 ± 3.6b	49.9 ± 2.6a	3.28 ± 0.47bc	5.10 ± 0.12b	29.9 ± 1.3b	31.5 ± 3.9abc
IHP	14.7 ± 0.3c	38.8 ± 3.9b	1.60 ± 0.11d	4.20 ± 0.53bc	12.2 ± 1.5d	37.7 ± 2.6a

Values are means of three biological replicates (SE). Statistical differences (*p* < 0.05) between OP treatments are indicated by different letters in each column.

**Table 4:** Influence of different phytin concentration supply on the total root morphology of barley.

Phytin Treatments	Length (cm)		Surface Area (cm^2^)		Average Diam (mm)		Volume (mm^3^)	
	CN4027	Baudin	CN4027	Baudin	CN4027	Baudin	Baudin	CN4027
−P	56.6 ± 1.6c	78.2 ± 3.8b	9.37 ± 0.24b	12.38 ± 0.56b	0.49 ± 0.04a	0.51 ± 0.03a	127 ± 7a	148 ± 13ab
LP	48.4 ± 2.4d	51.9 ± 2.1c	6.08 ± 0.50d	7.31 ± 0.53d	0.39 ± 0.01b	0.36 ± 0.01c	58.7 ± 6.7d	61.5 ± 1.8d
NP	36.9 ± 2.5e	50.9 ± 0.3c	4.86 ± 0.19e	8.74 ± 0.05cd	0.38 ± 0.01b	0.41 ± 0.01bc	46.7 ± 2.7e	67.3 ± 1.3d
H1P	64.7 ± 3.2b	72.8 ± 2.0b	8.23 ± 0.35bc	10.34 ± 0.30c	0.41 ± 0.00b	0.47 ± 0.04ab	83.7 ± 2.8c	142 ± 12b
H2P	58.2 ± 3.0bc	78.7 ± 2.2b	7.55 ± 0.53c	9.76 ± 0.52c	0.41 ± 0.01b	0.41 ± 0.03bc	72.5 ± 4.0c	93.7 ± 5.6c
H3P	80.7 ± 1.2a	122 ± 5a	10.6 ± 0.5a	17.5 ± 1.0a	0.42 ± 0.01b	0.46 ± 0.01ab	104 ± 2b	171 ± 10a

Values are means of three biological replicates (SE). Statistical differences (*p* < 0.05) between phytin levels are indicated by different letters in each column.

## Data Availability

All data generated or analyzed during this study are included in this published article and its Supplementary Information file.

## Supplementary Materials

The following supporting information can be downloaded at https://www.mdpi.com/article/10.3390/ijms242417191/s1.
