# Supplementary material for: The Impact of Various Organic Phosphorus Carriers on the Uptake and Use Efficiency in Barley
Source: Int J Mol Sci. 2023 Dec 6;24(24):17191. doi: 10.3390/ijms242417191 (PMC10743010; doi:10.3390/ijms242417191)
Supplement: Supplementary file 1 [file ijms-24-17191-s001.zip › ijms-2710380-supplementary.pdf]

Supplementary Material

Table S1. The specifc primer sequences for qRT-PCR

Gene	Forward sequence	Reverse sequence	Reference
Hv_GAPDH	5′-GCTCAAGGGTATCATGGGTTACG-3′	5′-GCAATTCCAGCCTTAGCATCAAAG-3′	Dai *et al.*, 2011
HvPAPhy_b	5′-AGATGACATTGGCTGTTGGTT-3′	5′-CGGAGGTGTAGTTCTGGAGG-3′	Dai *et al.*, 2011
HvPHT1;8	5′-GCCAACAAGCGCACAAG-3′	5′-TGAAGCGGTCGAACACG-3′	Developed in this study
HvPAP1	5′-AACTATACAGTGAACTGCAAGGAC-3′	5′-AATGGTAGCCAGCAAACAAG-3′	Developed in this study
HvPAP16;1	5′-ATTTGGGATGTCGGTGGTA-3′	5′-AGGGATTCGGAGCTGGA-3′	Developed in this study
HvPAP16;2	5′-CAGGTATAAGGAGAAGAGATGGG-3′	5′-GCTGTGGCAAAGAGGGAA-3′	Developed in this study
HvPAP16;3	5′-TCGCCTCTGTGGTGATGTAT-3′	5′-CTCTCGCAACGGTCTTGTT-3′	Developed in this study
